# Living with obesity — existential experiences

**DOI:** 10.1080/17482631.2019.1651171

**Published:** 2019-08-14

**Authors:** Venke Ueland, Bodil Furnes, Elin Dysvik, Kristine Rørtveit

**Affiliations:** a Faculty of Health Sciences, University of Stavanger, Stavanger, Norway; b Research Department, Stavanger University Hospital, Stavanger, Norway

**Keywords:** Obesity, phenomenological hermeneutics, lived experience, Kierkegaard, Merleau-Ponty

## Abstract

**Aims and objectives**: The aim was to gain in-depth understanding about individuals’ existential experiences of living with obesity.

**Background**: People living with obesity face great vulnerability and existential challenges. The different treatments offered do not seem to meet the individual needs of persons with obesity. A deeper understanding of existential experiences from an individual perspective is needed to individualize treatment.

**Design**: An exploratory phenomenological–hermeneutical design was used to gain a greater understanding of the existential experiences involved in living with obesity.

**Methods**: The participants represented a convenient sample. 18 qualitative interviews were conducted and subjected to phenomenological–hermeneutical analysis.

**Results**: Four themes emerged: *shaped by childhood; captured by food; depressed by the culture*; and *judged by oneself*.

**Conclusions**: The burden of being obese can be experienced as being *objectified* and *alienated* as a human being. We need to turn towards a life-world perspective, seeing *each human being as a living body* to overcome objectification and alienation, and then move them towards becoming subjects in their own lives, *through giving space for self-love*. Health care workers need to assist persons living with obesity to reduce objectification and alienation. It is important to develop intervention that has an individual, holistic approach.

## 1. Introduction

With a growing number of people living with obesity, health care has an obvious duty to be prepared to face their existential experiences. Despite the well-known health risks of obesity, the existential experiences of obese people are often overlooked by health workers (Overgaard, 2002). The World Health Organization (2018) has stated that obesity is one of the most pressing contemporary health challenges. A global obesity “epidemic” is associated with major implications for both individuals and for society (Grønning, Scambler, & Tjora, 2013; Hensrud & Klein, 2006; World Health Organisation, 2018).

Research indicates that obesity can reduce the quality of life, pose challenges to daily life, psychosocial well-being and self-esteem, and increase personal vulnerability (Christiansen, Borge, & Fagermoen, 2012; Overgaard, 2002). Individuals often report long and complex histories of weight gain in the development of obesity, related to family structures and early socialization experiences (Owen-Smith, Donovan, & Coast, 2014). Moreover, people with obesity are often confronted by stereotypical characterizations and stigma distributed through socialization, health care, media and language disclosure (Grønning, 2014; Pantenburg et al., 2012; Spahlholz, Baer, König, Riedel-Heller, & Luck-Sikorski, 2016). Persons suffering from obesity deal with health and therapy choices, which raise a complex set of questions without easy answers (Grønning, 2014). Health-care professionals seem to have adopted a reductionistic approach when caring for people with obesity. The body needs to have potential for improvement (Grønning et al., 2013). Available treatments such as surgery and lifestyle interventions make it difficult to find the optimum solution for the individual patient (Hofmann, 2016). After different types of obesity treatment, affected individuals still state they are struggling with their notions of self-existence (Rørtveit, Furnes, Dysvik, & Ueland, 2017). The current health services offered carry norms concerning the ideal body size and lifestyle, which tend to impose an objectifying and disciplinary control on the body (Jansen & Wehrle, 2018; Knutsen, Terragni, & Foss, 2011). Focusing on bodyweight and lifestyle change can be understood as a way of directing individuals towards what the culture sees as “normal” (Jansen & Wehrle, 2018; Knutsen et al., 2011).

Research has documented a wide range of contemporary perspectives connected to obesity (Curtis & Davis, 2014; Duarte, Pinto-Gouveia, & Ferreira, 2014; Hernandez-Hons & Woolley, 2012). Research often has a diagnostic approach using defining terms such as “binge eating” or “food addiction” (Curtis & Davis, 2014; Duarte et al., 2014). The concept of “food addiction” is increasingly employed in the academic literature, (Cullen et al., 2017).

## 2. Background

People living with obesity have typically been through a set of initial weight loss attempts, followed by weight gain rebounds, stagnation and resignation. As such, living with obesity seems to result in emotional distress (Bombak & Monaghan, 2017; Overgaard, 2002; Owen-Smith et al., 2014). This emotional distress can be reinforced by physical illness and disability, which further hinder participation in social- and health-related activities, leading to further emotional distress and ongoing weight gain (Owen-Smith et al., 2014). Studies report that people living with obesity describe an increasing culture of blame, amplified by media and public health messages (Bombak & Monaghan, 2017; Carryer, 2001; Thomas, Hyde, Karunaratne, Herbert, & Komesaroff, 2008). Given this struggle against weight gain and stigma, affected individuals also struggle with being perceived as obese and seeing themselves as obese (Christiansen et al., 2012). Seeing oneself as an obese person is a process that implies experiencing oneself as being significantly different from others (Christiansen et al., 2012). It is like living in a state of unease between hiding and showing one’s body, between being oneself and not being oneself (Overgaard, 2002). From a phenomenological perspective, body weight must be understood as an integral part of subjective meaning-making. Bodily self-awareness is tacit and pre-reflective (Merleau-Ponty, 2002; Rugseth & Standal, 2015; Svenaeus, 2011). Dismorphia may emerge when people experience bodily change as obesity, meaning that the seamless unity between body and world is disrupted (Leder, 1990; Rugseth & Standal, 2015; Zeiler, 2010). In this situation the body stands out as an object and a person might experience their fat body as “another” (Murray, 2007, 2010). Experiences of bodily dismorphia can be triggered by the views of others (Leder, 1990).

It might be claimed that the experience of bodily largeness is a health-threatening experience created by rigid, social and medical requirements that one should inhabit particularly small and artificially restrained bodies. It is disturbing to note that suggested treatments, combined with prolonged stigmatisation, might prove far more detrimental to health than simply having a large body (Carryer, 2001). In the end, feeling stigmatised is related to being different because of one’s body size, and consequently feeling not quite human (Merrill & Grassley, 2008). The person desired “image” of oneself incorporates the body and make the disconnect to oneself. The body might be at the forefront of all experiences (Murray, 2010). Depending on their bodily discomfort, individuals may experience disrupted intentionality and unhomeliness (Svenaeus, 2011).

Based on previous research, people living with obesity experience great vulnerability and existential challenges as human beings (Christiansen et al., 2012; Overgaard, 2002). Despite increased attention in practice and research on how to reduce stigma and stereotypical thinking, research shows that obese people still feel that they are less than fully human (Merrill & Grassley, 2008).

The different treatments offered do not seem to meet the specific needs of persons living with obesity (Grønning, 2014). There seems to be a neglect by health care of the lived embodiment. Medical science focuses on making bodies “normal”. The clinical view in medical discourse ignores tacit bodily knowledge (Murray, 2007). Despite wide research on the topic of obesity, promoting health and well-being for affected individuals appears to be difficult (Christiansen et al., 2012; Thomas et al., 2008). Moreover, there seems to be a gap between existing literature on changing habits and achieving weight loss, and getting successful results. Earlier research highlighted the need for developing more in-depth understanding of existential and interpersonal dimensions of living with obesity (Overgaard, 2002). To succeed in treatment, there is a need to understand the existential experiences of living with obesity, as a foundation for good practice (Overgaard, 2002).

For a human being, the lived body is the centre of orientation of themselves in the world. Therefore, we need competence regarding existential experiences. Consciousness about one’s lived body is essential to be able to think of oneself as an embodied unity (Jansen & Wehrle, 2018; Murray, 2010). Unity must be the primary approach for health care with a humanistic and holistic attitude (Knutsen et al., 2011; Natvik, Råheim, Andersen, & Moltu, 2018; Zeiler, 2010).

It appears there are few studies exploring what it is like to live with obesity as an existential challenge. As far as we know, only a limited number of qualitative or phenomenological studies have examined this aspect (Carryer, 2001; Christiansen et al., 2012; Merrill & Grassley, 2008; Overgaard, 2002; Owen-Smith et al., 2014). Based on the above considerations, we suggest that a deeper understanding of the existential experiences of obesity from an individual perspective is needed to provide more individualized treatment.

This study aimed at gaining an in-depth understanding of the individual existential experiences of living with obesity. The research questions we formulated were as follows: How does the person living with obesity experience her/his own body? How does the person living with obesity experience (being) herself/himself? How does the person living with obesity experience her/his life?

## 3. Methods

An exploratory phenomenological–hermeneutical design (Kvale & Brinkmann, 2009) was chosen, using qualitative interviews, to explore and interpret the existential phenomena related to living with obesity. Such an approach is advisable when the field of research is complex, requiring in-depth understanding. Further, this approach is useful for exploring a field with a limited research background and may be a key to gaining an understanding of the human life-world (Kvale & Brinkmann, 2009).

### 3.1. Data collection

The participants represented a convenient sample. They participated in a program directed by the specialist health service in 2016 and were invited to participate by the program leader. The interviews were not related to the actual program. The inclusion criteria were a BMI of 30 or more and obvious motivation to participate in an individual in-depth interview. The qualitative interviews lasted from 30 to 90 minutes.

The program leader informed 30 individuals about the research project and provided written information about the study. All those who decided to participate in the study made contact with the program leader. The first author then contacted the relevant participants and planned for an interview. Confidentiality was guaranteed and a written consent form was obtained prior to the interview. The participants gave their consent after receiving written information from the researcher. The project was approved by the Norwegian Ethical Committee (no. 2015/1720) and by the Norwegian Social Science Data Services. Participants were informed that they could withdraw from the study without giving any reason. Steps were taken to disguise the identity of the persons being interviewed. Participants had access to the support of professionals at the clinic if needed.

The sample comprised interviews with 18 participants (15 women and three men), all with a BMI in the range of 30–45 (see Table I). The participants reflected on life and existential situation living with obesity.

*Please talk about how you experience living with obesity?* Follow-up questions were aimed at uncovering thoughts and feelings in relation to their lives.

What is essential for you in your situation? What is challenging in life situations when living with obesity?

**Table I.** Participants (N = 18) background information.

Participants	Age	Occupation	Disorder
Female	33	-	Strain injuries
Female	64	Retired	High blood pressure, strain injuries
Female	50	On sick-leave	Low metabolism, strain injuries
Female	44	Receiver of disability benefit	Low metabolism, strain injuries
Female	45	Working	-
Female	47	Working	Strain injuries
Male	71	Retired	Strain injuries, breathing problems
Female	42	On sick-leave	High blood pressure, strain injuries
Male	57	Receiver of disability benefit	Strain injuries
Female	31	Working	High blood pressure, strain injuries
Female	49	Working	Strain injuries
Female	50	On sick-leave	Strain injuries
Female	58	Working	High blood pressure, strain injuries
Female	63	Retired	High blood pressure, strain injuries, diabetes
Female	51	Working	Low metabolism
Female	24	Unemployed	Strain injuries
Male	46	Working	Depression, breathing problems
Female	51	Working	Low metabolism, bipolar disorder

Central to the approach was openness towards the subjects’ experiences and descriptions of the research topic, with an attempt to disregard prior knowledge and search for descriptions of key importance. The in-depth interview focused on the person’s life story related to obesity and her/his existential situation. The researcher was careful not to violate the integrity and dignity of the participants. Data were audio recorded and transcribed verbatim by one of the researchers.

### 3.2. Data analysis

Interpretation of texts was guided by Kvale and Brinkmann (2009) and analyses took place at three levels: self-understanding, common sense and theoretical understanding; see Table II. The primary author drafted the analysis. All editors were involved in discussions throughout the process of interpretation.

**Table II.** Examples of three contexts of interpretations—thematised findings.

Three contexts of interpretations		
Self-understanding	Common sense	Critical common sense
*I feel a kind of hopelessness. Shame and despair are connected here. I think that it started when I was born, so it’s an extensive part of my personality*.	*Being shaped by childhood*	The person feels self-blame and self-rejection, which may feel like being *objectified* as a human being (Kierkegaard/Merleau Ponty). The person isolates herself/himself from relationships with others, which may feel like being *alienated* as a human being (Kierkegaard/Merleau Ponty).
*The food has trapped me. It has made some deep traces. I don’t bother losing weight, I continue eating*,	*Being captured by food*	
*I hear it every day. Every single day someone talks about overweight*.	*Being depressed by the culture*	
*I see myself as terribly thick and chubby and as one who has failed.*	*Being judged by oneself*	

The process between understanding and interpretation occurs as a hermeneutical movement between the particular, the universal and the whole part (Gadamer, 2007). According to Gadamer’s philosophy, the interpreter cannot be fully aware of his/her own prejudices and pre-understanding. To interpret is to ask questions such as: “*what is it like to live with obesity*” and “*how do people suffering from obesity experience life*”.

#### 3.2.1. Self-understanding

The interpretation started by the authors reading through all the interview texts to grasp an intuitive first holistic understanding. Through these readings, fragments of something significant in relation to the lived experience of living with obesity emerged. The first stage of interpretation is to reveal the self-understanding of the participants. Units of meaning were collected, and small headings were inserted in parts of the texts. Units of meaning belonging together were examined and arranged under preliminary headings.

#### 3.2.2. Common sense

The second step refers to comprehension of the interview text within the context of a reasonable, universal understanding. New themes with a new level of abstraction came forth by reformulating the participant’s self-understanding. The units were systematized in closely connected topics, with the new level of abstraction giving the four themes presented in the Results section. To enhance the rigour of the interpretation process, all preliminary themes were discussed between the authors and reformulated until consensus was achieved. The first two levels of interpretation (self-understanding and common sense) are also presented in the Results section.

#### 3.2.3. Theoretical understanding

The first two levels provide an indication of relevant theory that might enrich the discussion (Kvale & Brinkmann, 2009). The third level of interpretation is a theorization based on other research and relevant theories, which may illuminate and deepen the understanding of findings. Theoretical interpretations are helpful to achieve in-depth understanding. Theory serves to extend the sources of knowledge beyond the empirical data (Malterud, Siersma, & Guassora, 2016). This level of interpretation is presented in the Discussion section.

## 4. Results

Thematised results are: *shaped by childhood, captured by food, depressed by the culture*, and *judged by oneself*. These themes represented the participants’ descriptions of living with obesity. They described being deeply challenged by society, by memories from childhood; they felt captured in a deep furrow and by their own self-blame.

### 4.1. Shaped by childhood

The participants described how their relationship with food had already been shaped in childhood. Several informants knew perfectly when their overweight problems started. Food and meals seem to have secondary functions such as restoring the atmosphere at home and compensating for lack of an intimate relationship.

—That I know really well. It was at home when I was a child.

Some of them pointed to a problematic relationship to meals and eating during childhood. They found that eating was controlled by the adults:

—The portions we were given were too small and we were not allowed any food after 5 o’clock. When I went into the kitchen trying to sneak some food my mum would notice it and yell ‘No more food!’ After I moved out, I binged and puked for many years.

Another form of disruption was associated with an atmosphere of conflict in relation to food and meals. This was described by one participant:

—My mother ruined the pleasure of food when I was a child. She had such a crazy relationship with food. If anything bad happened, food would fix it!

—There were recurrent conflicts around the table about the amount of food. I never got an easy relationship with eating. There was no self-regulation of meals.

Several participants reflected on the link between their relationship to food as adults and the absence of intimacy in their childhood, the lack of approval and the experience of rejection. One of them recounted a hospital stay when she was little:

—My parents were not allowed to be there. This has left a lasting impression. I have major issues with intimacy. Food has become a compensation for the lack of closeness.

Some participants felt comforted by eating. When closeness to others was lacking, food provided some closeness to themselves, which gave a feeling of comfort. In this sense, food soothed pain, grief and loss, and concealed vulnerability. One interviewee dwelled on her childhood and adolescence. She believed that the pattern of overweight began in the early stages of infancy.

—I feel a kind of hopelessness, where shame and despair are interconnected. I think it started when I was born, so it’s an extensive part of my personality. How am I supposed to change that? It’s a self-fulfilling prophecy.

Participants described how food and meals came to be something profoundly personal; a sensitive area of what it meant to be human. Hunger and satiety seemed to be a personal part of life. Judging from the interviews, it appeared as if their childhood memories made it difficult to look for opportunities for change. The understanding of oneself and one’s eating pattern and overweight became self-fulfilling. This seemed to be challenging when distinguishing their own needs and asking: *who am I*, and *what do I feel and need*? Basic perceptions of hunger and satiety appeared to be disturbed or destroyed, and it became difficult to become self-consistent.

### 4.2. Captured by food

Several participants described being trapped by food, reflecting on the difficult psychological issues related to this process. Their relationship with food had taken a major role in their life and the path to change seemed difficult:

—The food has trapped me. The psychological part is the hardest. It has been a long process to acknowledge that it’s all in my head. It has made some deep marks. I don’t bother to lose weight, I continue eating. I’m living in a bubble and I can’t break out and turn the knowledge I have into action.

The expressions “trapped” and “living in a bubble” expressed that the participants were unable to live the life they wanted. The traces being formed were restrictive to being able to live a free life instead of being controlled by food. Some were struggling to understand themselves in the light of a diagnosis of eating disorders and addiction. Others felt they did not fit into such descriptions:

—First, I had an alcohol problem, then I think I traded the alcohol for food. There is something compulsive about my relationship with food. It’s pretty stupid, but I still end up taking another piece of that cake.

—There’s something uncontrollable about taking that piece. I don’t see myself as someone with an eating disorder, more like someone who’s preoccupied with food in an unhealthy way. If I start counting calories, I quickly feel hostile about food. I think vulnerability and pattern are better words than addiction, and for me vulnerability is the most important.

The reflections of the informants were coloured by a feeling of being “captured by food”, a variation to understanding themselves. Their obesity pointed at a psychological trap. The explanations they gave implied that living with obesity they were stuck in dimensions that prevented the changes they desperately wanted. However, terms such as “vulnerability” could open up the road ahead.

### 4.3. Depressed by the culture

Several participants stated they were judged by the culture, not only for their bodies, but also because of the amount of what they ate. They perceive themselves as constantly observed and devalued by their acquaintances. The relationship they have with food has become visible:

—At work they talk about food in every context. It’s completely twisted. Being fat, I know perfectly well what’s wrong. All this talk about food only increases my self-reproach.

—I’m a member of many enjoyable women’s clubs. When food is served I need to be careful and help myself with moderation. But when food is put on the table … I tend to become nervous about eating too much, and then it becomes a self-prophecy. I imagine them saying: “But she’s so fat! Why does she take that piece of cake, shouldn’t she refrain from it?” The whole thing is so internalised. But who would give you permission, what is it that gives you this self-reproach, it has a lot to do with society, the focus on training and losing weight.

The participants were stressed by the idea of being expected to fit in, and not exceed the norm. They reflected that society controls food and eating, and that it defines standards on how the body is supposed to look. Further, they suggested that the mirror put up in front of them by community and culture could be internalised easily, becoming the way in which they looked upon themselves and their body.

—It tears at my self-esteem. The way others look at me. I compare myself with the rest of my family. They are of average weight and fine. I’m an outgoing personality, but I notice that I decline invitations when I’m at my worst. I don’t even have a dress. People don’t stop inviting me, but I choose to withdraw myself more often.

Several participants emphasized being affected both directly and indirectly by public criticism of obesity. It impacted on how they lived their social lives, restricting their freedom and participation in social events. In this way, the voice of culture restricted their self-realization. To see oneself as different, being unable to reach the expectations and standards of the surrounding environment had consequences. It gave them a poor self-image and made them feel aggression towards themselves and others.

—I hear it every day. Every single day someone talks about overweight. I’m going to a party on Saturday and I know someone is going to comment on it. I always feel like everyone pays attention to how much I eat.

—It’s the same old story; big people are stupid, because grabbing an extra piece of cake is stupid. Sometimes I think, who cares, it’s not their business.

Prejudgement took over and gave a feeling of self-hatred, shame and guilt, as they felt less worthy than others. This made it harder to shield themselves from the voice of culture because they are part of it. It meant that they internalized their feelings, which led to self-judgment.

The participants’ statements show how deeply society affected them as human beings. They said they looked on themselves with the eyes of society, as if they had adopted the voice of society. They reflected on not being able to hide their body, as it related to the outside world and on being judged for being too big. As well, they reflected on being captured in society’s and culture’s prejudgement. The community’s relationship with food lay like an outer pressure, a grip around them.

### 4.4. Judged by oneself

The participants discussed how their self-judgement was based on their recognition of their own body. For example, they said they would try to conceal their stomach in social settings and avoided pictures being taken of their whole body. They disowned their bodies, which had become bodily objects. The idea of appearing obese led to a patronizing feeling of self.

—I see myself as terribly thick and chubby and as a failure even if I have not really failed at all.

—I see it as a weakness. If I were strong enough, I would not be where I am.

Participants described how such feelings and thoughts about themselves led to feelings of weakness and self-blame for being wrong. Several of them expressed that they longed for another life, as they missed the joy of life, and of having to struggle with self-blame and self-hatred.

—When I over-eat I get immense self-contempt. At times I can sit up all night waiting to go to bed, just to be done with the day, I don’t feel depressed, more unmotivated for life.

—The joy of life and pleasure of food are destroyed for me. I have sought help, I have tried to make it, but I’m stuck in that bubble. I have resources and I attend courses. I’m always trying. I hardly ever give up, but I think: Why can’t I live peacefully for the rest of my life, instead of stressing with this. I don’t know if I’ll be able to cope any longer.

Caught in a conflict between losing weight and not being able to, carrying themselves and their weight, they felt persisting pressure. It was as if they had lost a part of themselves, the joy of life and the opportunities for self-expression. They saw no options for moving on. Some said that the lack of joy and enthusiasm had made them strangers to themselves, because of their lack of vitality. Several of them said they were close to giving up. Their experiences of guilt, shame, self-blame, anger and hopelessness were clearly communicated.

Participants looked for ways out from their self-judgement and the tracks in which they were captured. They described how they worked with their thoughts and feelings, pondering on how they could in the best way possible move on.

—The kids say I’ve become grumpy, angry and cranky, whereas I used to be so cheerful. Why can I not get this sorted out? I haven’t taken care of myself! I have been judging myself. If even my mother cannot give me recognition and empathy, how am I supposed to see myself in a positive light and acknowledge myself? Yes, the key is probably in acknowledging yourself, that makes sense.

—I felt it when I was going to set goals, today I’m sitting here crying because I’m going to tell you what those goals are.

The empirical findings underline that those who live with obesity creates a firm, hard and degrading tone towards themselves. When the interview centred on the participants, it touched something deep inside them. Their self-pity hit a sore spot. Being the centre in their own life, acknowledging themselves, seemed to touch the core of their vulnerability in being obese.

## 5. Theoretical interpretation and discussion

The aim of this study was to gain an in-depth understanding about individuals’ existential experiences of living with obesity. From our interpretation of the concepts of *being shaped by childhood, captured by food, depressed by the culture*, and *judged by oneself*, we suggest that the burden of being obese is experienced as being *objectified* and *alienated* as a human being. In the following, the concepts of being “*objectified* and *alienated*” form the structure of the discussion. The overall interpretation makes use of Merleau-Ponty’s rigorous philosophy of the body and the philosophical thinking of Kierkegaard about what it means to be human and how to become one’s self.

### 5.1. To be objectified as a human being

Our findings illuminate the idea of being objectified as a human being. Living with obesity leads to thoughts and feelings of *being depressed by the culture*. At an existential level, this can be understood as being exposed to outer and inner objectification (Malterud & Ulriksen, 2010; Shaughnessy, 2018). More commonly, outer objectification can be related to experiences of struggle with the cultural expectations of slimness, and of a person being scorned for their visible body. The inner objectification is subsequently an internalisation of stigmatisation.

Being stigmatised gives one a feeling of being constantly observed, an awareness of not being seen as a subjective human, but as an object. Limiting social contact is a way of avoiding the risk of receiving remarks about body size for obese people. In line with French philosopher Merleau-Ponty, humans naturally experience themselves as both subjects and objects in their lives. He describes how a person’s embodiment may become broken when he/she experiences life-changing events, such as illness. Our findings suggest that living with obesity represents such brokenness. When persons have an increased awareness towards the failing obese body, the seamless unity between the body as both subject and object, might be disrupted (Merleau-Ponty, 2002). The increased consciousness about ones obese body seems to make a split between the subjective and the objective body. The experiences of the lived body might change from “I am a body” to “I have a body” (Merleau-Ponty, 2002). When immersed in an enlarged body, an immediate way of life can be lost. Merleau-Ponty (2002) stated: “In so far as I have a body, I may reduce [myself] to the status of an object beneath the gaze of another person, and no longer count as a person for him” (p193).

From our study, we found that a person’s obesity tends to lead to inner objectification through self-judgement, which is illuminated by the theme *being judged by oneself*. Bodily self-judgement again leads to an urge to hide one’s body and oneself, to avoid being exposed. In other words, almost an alien’s perspective of one’s own body. Orbach (2011) claimed that in today’s society, the body often appears as an object, and not something we *are*. Moreover, commercialisation of the body exposes everybody to basic shame and despair of oneself (Orbach, 2011). Orbach (2011) underlined that something outside us highlights the body as an object. This view of one’s body affects our identity and becomes part of who we are.

Our study clearly shows that becoming the subject in one’s own life, can be a struggle. The relation the person has with oneself seems to be the primary challenges. Rugseth and Standal (2015) sites from an article of Murray: “… my fatness emerges as a barrier between myself, and my body, between myself and others, rather than being the ‘very horizon’ that brings me into being” (Rugseth & Standal, 2015, p. 8)

Kierkegaard state that despair is a form of misrelating to one self. The individual begins to regard him or herself as something different, something that does not necessary fit into the given cultural settings (Nielsen, 2018).

The findings show that obese people can’t go about their life hindered by their large body. It is about the self-relation of the person, more than about not being able to participate in daily life activities living a “normal life”. Kierkegaard (1849) claims that it is challenging for human beings to become themselves. This is probably what is experienced by persons living with obesity; they have a limited opportunity to live out their potential for life.

Several studies confirm the cultural pressure that exists towards persons living with obesity (Overgaard, 2002; Owen-Smith et al., 2014; Thomas et al., 2008). The stigma of body size is an attribute that distinguishes its bearer as being different from what society defines as “normal” (Grønning et al., 2013). An inner process towards objectivising can occur when the participants look upon their body merely as a failure and a burdensome physical object needing improvement. This internal process creates a distance to the body, and further a distance to one self as a unity (Merleau-Ponty, 2002).

Our findings suggest that the participants have been caught in a vicious circle of food, eating and inability to lose weight. To possess knowledge and self-awareness, without being able to create the desired change, leads to self-blame, which further makes them objectivised; this leads to *judging oneself*. It appears that the body needs to be disciplined to an excessive extent as it becomes a potential for improvement (Grønning et al., 2013). When the body rejects being controlled, shame and blame can occur (Grønning et al., 2013). This is in accordance with the idea that the body has become a dynamic site of aspiration in its own right, constantly working on the site (Grønning et al., 2013). Multiple failing weight control strategies can lead to obese people assigning personal self-blame (Malterud & Ulriksen, 2010). Struggling with weight loss can expand the distance from one’s own body and suppress the self, one might be struggling with being one self. According to Kierkegaard, conforming to a representative, cultural ideal is to loose one self (Nielsen, 2018). Further, he states that becoming oneself always begins through knowing and accepting oneself in the present (Batchelor, 2006; Kierkegaard, 1849). Gaete and Fuchs (2016) claimed that the public health service seems to enhance such objectification of the person’s body. Health services focusing, for example, on lifestyle-changing programs might increase attempts to control the obese body, but this can also lead to an increased objectification of the body (Grønning et al., 2013). Gaete and Fuchs (2016) confirmed that it appears to be difficult for people struggling with weight problems to stay in a subject position, as their lived body is in a vulnerable and unbearable state. Therefore, it must be an important therapeutic goal to look behind the symptoms if possible, to make a shift of rigid objectification of one’s own body towards a subject position (Gaete & Fuchs, 2016).

As described here, a person living with obesity experiences being *captured by food*, which feels like being trapped in a deep space or living in a bubble. Using terms such as “addiction” or “diagnosis” is neither helpful nor adequate to describe what they experience, and such diagnoses are in some cases problematic. The diagnosis might be felt to be too dominant, and might in itself become an explanation that prevents change. The terms “addiction” and “eating disorder” are diagnostic and typically used to objectify the condition. For some, this may be a help to understand themselves, whereas others do not need diagnostic terms. We believe it would be better to reflect on vulnerability and eating patterns to break the negative pathway. This indicates that the use of a diagnosis disturbs a potential success route to weight loss. It can also objectivise and suppress the individual.

Conflicts and longing for enjoyment of food came to light in the theme *being captured by food*, and show how powerful the unfortunate relationship with food can be. Meals are a sort of relief and purification after disturbance, and eating carries repercussions after an adverse incident. A fundamental, natural and effortless relationship with food and meals is disrupted by such childhood experiences. “Living one’s body” (Merleau-Ponty, 2002) might be equivalent to living one’s life eating, in some way living a life through meals and food. As described here, the meal loses its intrinsic value, becoming something that cannot be enjoyed, rather than something that is necessary and nice.

### 5.2. To be alienated as a human being

*Being shaped by childhood, captured by food, depressed by the culture*, and *judged by oneself*, point to a feeling of being in a hostile or indifferent state of mind towards oneself (Merrill & Grassley, 2008). The awareness of not being regarded as a person can create alienation from oneself (Barber, 2016). As well, the experiences from one’s own *childhood, food, culture* and *oneself* can create a distance to one’s own body and lead to an objectivised way of being. Merleau-Ponty (2002) postulated that humans both *have* a body and *are* a body, they are *living one’s body*. As such, we experience existence through the body. The experience of oneself, one’s body and one’s presence in the world precedes reflections and is the basis for experiencing the life-world (Merleau-Ponty, 2002). Being alienated from both society and oneself appears like an existential homelessness, as not living in one’s own body fully.

To be a human being in becoming towards wholeness, is to make space for oneself and what is given by life, according to Kierkegaard. This is to be transparent towards one self and choosing oneself (Cruysberghs, 2010). Our study shows that the body seems to be separated from one’s self and seems to becomes an attachment to one’s self. Self-deception hinder the person from being a healthy unity. Self-knowledge includes a painful process of understanding oneself and facing the ambivalence to one self (Welz, 2011a). The despair on the other hand might be not to be willing to be oneself, and then hiding oneself (Welz, 2011a). “One does not want to be who one is, while one at same time cannot get rid of oneself” (Welz, 2011a, p. 179).

Kierkegaard believes that owning one’s own heart, one’s brokenness, is only possible when resting transparently in the power that established it (Cruysberghs, 2010; Kierkegaard, 1849). Standing before the second person (God) is what brings this home (Lippitt, 2017).

Past experiences are part of the lived body in time and space (Merleau-Ponty, 2002). The findings in our study, *being shaped by childhood*, disclose a self-understanding of how the obesity is strongly related to the person’s history. Early childhood experiences and challenging events in life lie under the surface. Thus, causes of obesity are related to early childhood, such as loss of significant persons, divorce or illness. Stories from childhood about eating and meals as a scene of struggle are highlighted as reasons for the current relationship to food. It seems as if seeing one’s own obesity in relation to memories holds the person trapped in hopelessness and self-blame. By linking one’s own obesity to past experiences, the state of obesity becomes fundamental, seemingly impossible to change, and make oneself alienated. It appears as a self-fulfilling prophecy. This is in accordance with other studies that examined weight-related challenges dating back to adolescence (Malterud & Ulriksen, 2010; Ritten & LaManna, 2017). Hofmann (2016) found that food and eating are phenomena with strong cultural bearings and that they are profoundly private. By relating to a fixed past, humans can feel they are victims of circumstances. Being in control of the circumstances may limit their freedom through disownment of their lived body (Barber, 2016).

Deprivation of liberty was evident in our study as a sense of being alienated from fully living one’s life. The theme *judging oneself* is related to feelings of shame about the body: labelling one’s own body as terrible, thick, chubby and ugly and feeling one’s obesity as a weakness. Because of shame about one’s body image, obese individuals may perceive themselves as unattractive, defective and rejectable. Thus, the body seems to be in a state of constant embarrassment, a negative place of disappointment.

Fuchs (2002) think with Merleau-Ponty and make a difference about the corporal body and the lived body. Lived body, is my embodied being-in- the- world. The corporal body are often looked upon as a mere result of objectifying reflection of the body, as a foreign body. The body can be an obstacle for living one’s life and limit the access to the world. The corporal body is thrown back on itself, connected with alienation, disappointment, embarrassment (Fuchs, 2002).

Fuchs states: ‘in the experiences of heaviness … the lived body—loses its “taken for granted” … and becomes the sluggish, obstinate or fragile body which I have” … “than the body loses its prereflective … and the spontaneous bodily expressions are disturbed, blocked, or objectified by an inversion of our attention upon ourself” (Fuchs, 2002, p. 224, 225).

Welz (2011b) pointed to shame processes such as struggling with questions of who one is, who one wants to become, and how one wishes to be seen. Experiences of shame can shrink the life-world and challenge one’s worth as a human (Merleau-Ponty, 2002; Welz, 2011b).

Studies on eating disorders highlight the tendency towards self-criticism and attacks on the self, as maladaptive ways of dealing with a body image of shame (Malterud & Ulriksen, 2010). Our findings about being *captured by food, depressed by the culture*, and *judged by oneself* indicate a tendency for the respondents to doubt and blame themselves for their own weakness. When one’s own body lets one down, somehow betraying oneself, the opportunity to find personal space, care for, and love oneself seems to be lost. That is experienced as being alienated as a human being, and may lead to loss of oneself. Kierkegaard (1847) pointed towards human beings struggling with love and self hate which point towards alienation of oneself (Kierkegaard, 1847). Lacking the abilities for warmth, kindness, self-compassion and love of self can pose challenges for persons living with problematic weight (Duarte et al., 2014). Being depressed by the culture and judged by oneself may be synonymous with what Kierkegaard describes as an experience of self-rejection (Lippitt, 2013). Alienation might be when love of self is weak and completely owning one’s place can be challenging. Experiencing brokenness in life, such as obesity, can release a struggle for self-preservation. Kierkegaard claims that despair can be the road to deeper reflection and to let go of established understanding of oneself (Kierkegaard, 1849). Kierkegaard (1849) suggested that love of self is a duty, and claimed that when one abandons the truth of the past, one may gain the freedom to live one’s life and break free from isolation. Struggling for self-love might be to open an interpersonal space and reduce self-devaluation, through reflection without judging oneself (Fuchs, 2002; Gustin, 2017). This seems to provide an opportunity for expanding the space for oneself, and for recognizing the worth of the self, which only love of self can give birth to (Lippitt, 2013).

## 6. Conclusion

Our findings have demonstrated the relationship between brokenness and “closing down” one’s life-world. Participants experienced living with obesity as an objectification and alienation from themselves and the world. Our findings suggest that traditional health services can lead to increased objectification. Health personnel need to turn towards a life-world perspective. Reducing objectification and alienation might be conducive to achieving more effective treatment. The possibility of obtaining weight loss and a more enjoyable life might be achieved when the person suffering from obesity overcomes objectification and moves towards becoming a subject in her/his own life, through love of themselves.

### 6.1. Implications for practice

We suggest that a deeper understanding of the existential experiences from an individual perspective is required to improve treatment. Obviously, the different treatments available for obese people in health care require a more holistic approach. Our findings might fill a gap caused by lack of in-depth understanding of objectification and alienation. When health care can accommodate the individual’s existential experiences, they can be empowered and find their own way. Persons need help to become aware of their own objectification of their body and the resulting alienation. First, they need to accept themselves as a whole “lived body”, to think of themselves as an embodied entity (Murray, 2010). To enable them to accept themselves as subjects, we suggest that one-to-one counselling is needed, where they can share the existential experiences of suffering from obesity. Through such reflections, they might be helped to become closer to their own body.

A phenomenological understanding of these existential experiences can inform professionals who deal with health issues related to persons with obesity. Health care workers, together with affected individuals, need to reflect on the existential experiences of living with obesity. Instead of meeting persons through diagnostic and classification labels, it is appropriate to rethink narrow definitions and seek instead to meet the person’s unique experiences, focusing on the person’s life-world. Our findings suggest that when persons living with obesity are liberated from objectification and alienation they are better prepared for working towards effective weight loss. In-depth knowledge from the sufferer’s perspective, might better guide health care workers on how to meet the person struggling with obesity, so that they can feel more human and achieve health.

### 6.2. Methodological considerations

The evaluations were based on criteria such as credibility, coherence and universality (Kvale & Brinkmann, 2009). It is a strength of the study that there is a relatively large number of participants (18), and we consider that we have received rich data. The researcher established a good dialogue with the participants and obtained rich descriptions, with both variances and deviances addressing the research question. The power of the information seems to be strong, so the number of participants might be more than sufficiently large and variable to achieve the aim of the study (Malterud et al., 2016). Universality was achieved through the study purpose, which was to arrive at a deeper and new understanding of the topic.

It is important to recognize the limitations of the study. We are aware that a convenient sample necessary will not give the richest information sources, although the participants were available regard to the themes, excess, location, time and willingness. Another limitation may be that the data could be influenced by the participant’s involvement at the time they were participating in rehabilitation, when they might focus on what they wanted to change in life. In our view, there are no general “lived experiences” of living with obesity, although there might be some common themes. Nevertheless, using multiple participants, we found a good deal of consensus as to how the person living with obesity experiences life.

As this was an explorative study, we did not aim for complete descriptions of all aspects of living with obesity, but we believe we have contributed substantially to in-depth knowledge about a challenging health problem (Malterud et al., 2016).
